# Reviews

**Published:** 1863-01

**Authors:** 


### REVIEWS.

*The Action of Medicines in the System; or, "on the mode in which Therapeutic agents introduced into the Stomach produce their peculiar effects on the Animal Economy." Being the Prize Essay to which the Medical Society of London awarded the Fothergillian Medal for* 1862; *by* FREDERICK WILLIAM HEADLAND, M. D., B. A., F. L. S., *Licentiate of the Royal College of Physicians, etc., etc. Fourth American Edition. Philadelphia:* LINDSAY & BLAKISTON, 1863.

CONTENTS.—Chapter I: Introductory Remarks.

Chapter II: On some of the more important Classifications of Medicines, and Opinions of Authors respecting their Actions.

Chapter III: On the General Modes of Action of Therapeutic Agents introduced into the Stomach; treated of in ten Propositions.

Prop. 1.—That the great majority of medicines must obtain entry into the blood, or internal fluids of the body, before their action can be manifested.

Prop. 2.—That the great majority of medicines are capable of solution in the gastric or intestinal secretions, and pass without material change, by a process of absorption, through the coats of the stomach and intestines, to enter the capillaries of the portal system of veins.

Prop. 3.—That those medicines which are completely insoluble in water, and in the gastric and intestinal juices, cannot gain entrance into circulation.

Prop. 4.—That some few remedial agents act locally on the mucous surface, either before absorption, or without being absorped at all. That they are chiefly as follows:

*a.* Irritant Emetics. *b.* Irritant Cathartics. *c.* Superficial Stimulants, Sedatives, Astringents.

Prop. 5.—That the medicine, when in the blood, must permeate the mass of the circulation, so far as may be required to reach the parts on which it tends to act. That there are two possible exceptions to this rule:

*a.* The production of sensation or pain at a distant point. *b.* The production of muscular contraction at a distant point.

Prop. 6.—That while in the blood the medicine may undergo changes, which in some eases may, in others may not, affect its influence. That these changes may be—

*a.* Of Combination. *b.* Of Reconstruction. *c.* Of Decomposition.

Prop. 7.—That the first class of medicines, called Hæmatics, act while in the blood, which they influence. That their action is permanent.

1. That of these some, called Restoratives, act by supplying, or causing to be supplied, a material wanting; and may remain in the blood.

2. That others, called Catalytics, act so as to counteract a morbid material or process; and must pass out of the body.

Prop. 8.—That a second class of medicines, called Neurotics, act by passing from the blood to the nerves or nerve-centres, which they influence. That they are transitory in action.

1. That of these some, called Stimulants, act so as to exalt nervous force, in general or in particular.

2. That others, called Narcotics, act so as first to exalt nervous force, and then depress it, and have also special influence on the intellectual part of the brain.

3. That others again, called Sedatives, act so as to depress nervous force, in general or in particular.

Prop. 9.—That a third class of medicines, called Astringents, act by passing from the blood to muscular fibre, which they excite to contraction.

Prop. 10.—That a fourth class of medicines, called Eliminatives, act by passing out of the blood through the glands, which they excite to the performance of their functions.

Chapter IV: On the Action of some of the more important Medicines in particular.

This book is so well known to the profession that we have not felt called upon to speak of its merits at great length. We have copied its general contents that students and practitioners may observe the points it discusses. We earnestly recommend it to the consideration of the profession, and advise its careful perusal. Great differences of opinion have always prevailed as to the positive and comparative value of almost all therapeutic agents; these differences must continue, and perhaps increase, while the faith of some may grow cold as to the value of many of the articles of the materia medica in common use. The theory of the action of medicines and the opinions of authors respecting it, as presented in this book, are full and highly interesting, and the book as a whole, without stopping to particularize, s well worthy the careful perusal of both physicians and students of medicine.

